# Genetic relatedness and virulence potential of *Salmonella* Schwarzengrund strains with or without an IncFIB-IncFIC(FII) fusion plasmid isolated from food and clinical sources

**DOI:** 10.3389/fmicb.2024.1397068

**Published:** 2024-05-17

**Authors:** Monique A. Felix, Danielle Sopovski, Seth Commichaux, Noah Yoskowitz, Nesreen H. Aljahdali, Christopher J. Grim, Carter N. Abbott, Ashlyn Carlton, Jing Han, Yasser M. Sanad, Shaohua Zhao, Xiong Wang, Steven L. Foley, Bijay K. Khajanchi

**Affiliations:** ^1^National Center for Toxicological Research, U. S. Food and Drug Administration, Jefferson, AR, United States; ^2^University of Arkansas at Pine Bluff, Pine Bluff, AR, United States; ^3^Center for Food Safety and Applied Nutrition, Office of Applied Research and Safety Assessment, U. S. Food and Drug Administration, Laurel, MD, United States; ^4^Department of Biological Science, Faculty of Science, King Abdulaziz University, Jeddah, Saudi Arabia; ^5^Center for Food Safety and Applied Nutrition, U. S. Food and Drug Administration, College Park, MD, United States; ^6^Department of Epidemiology, College of Public Health, University of Arkansas for Medical Sciences, Little Rock, AR, United States; ^7^Center for Veterinary Medicine, U. S. Food and Drug Administration, Laurel, MD, United States; ^8^Minnesota Department of Health, St. Paul, MN, United States

**Keywords:** *Salmonella* Schwarzengrund, IncFIB-IncFIC(FII) plasmid, fusion plasmid, SNP, conjugation, Caco-2

## Abstract

A total of 55 food and clinical *S*. Schwarzengrund isolates were assayed for plasmid content, among which an IncFIB-IncFIC(FII) fusion plasmid, conferring streptomycin resistance, was detected in 17 isolates. Among the 17 isolates, 9 were food isolates primarily collected from poultry meat, and 8 clinical isolates collected from stool, urine, and gallbladder. SNP—based phylogenetic analyses showed that the isolates carrying the fusion plasmid formed a subclade indicating the plasmid was acquired and is now maintained by the lineage. Phylogenetic analysis of the plasmid suggested it is derived from avian pathogenic plasmids and might confer an adaptive advantage to the *S*. Schwarzengrund isolates within birds. IncFIB-IncFIC(FII) fusion plasmids from all food and three clinical isolates were self-conjugative and successfully transferred into *E. coli* J53 by conjugation. Food and clinical isolates had similar virulome profiles and were able to invade human Caco-2 cells. However, the IncFIB-IncFIC(FII) plasmid did not significantly add to their invasion and persistence potential in human Caco-2 cells.

## Introduction

*Salmonella* is an enteric pathogen that invades the gut through contaminated food (Hallstrom and McCormick, 2011; Fabrega and Vila, 2013). They are Gram-negative, facultative anaerobes with food animal reservoirs, such as chickens, cows, turkeys, and pigs (Andino and Hanning, 2015). There is an estimated more than 1 million human cases of *Salmonella* infection each year in the U.S. with approximately 20,000 hospitalizations and 400 deaths (Scallan et al., 2011). *Salmonella* infections can be classified as either typhoidal (human specific), or non-typhoidal Salmonellosis (broad host range; Aarestrup et al., 2007). A significant rise of the *Salmonella* Schwarzengrund serovar in countries such as Thailand, Slovakia, New Zealand, Venezuela, the U.S., Japan, and Denmark has been reported (Aarestrup et al., 2007). For example, an increase in *S.* Schwarzengrund infections in humans from 0 to 2.4%, and in chickens from 0.3% to 26.2% from 1993 to 2001 was observed in Thailand (Bangtrakulnonth et al., 2004). Similarly, in Japan, of the serovars collected from broiler chickens, *S*. Schwarzengrund was found to account for 28.1% of isolates in 2005 compared to 0% in 2000. Asai et al. (2009) also reported an increasing trend of isolation of *S*. Schwarzengrund from human patients in Japan. Their findings showed that in just 2 years *S*. Schwarzengrund went from an uncommon serovar to the 10th most commonly reported. A more recent study, on 3,069 cecal samples collected from broiler chickens in a processing plant in Japan from 2013 to 2016, reported 17.8% were positive for *Salmonella* and 21.3% of those were identified as *S*. Schwarzengrund. The prevalence of *S*. Schwarzengrund had increased from the 2.1% reported in their previous study from 2009 to 2012 (Duc et al., 2020).

In the U.S., *S*. Schwarzengrund has risen to become one of the top five *Salmonella* serovars isolated from retail meat (Aarestrup et al., 2007). For example, in 2019, a *S*. Schwarzengrund outbreak linked to ground turkey occurred in three states and resulted in 78,000 pounds of turkey being recalled [Center for Disease Control and Prevention (CDC); https://www.cdc.gov/salmonella/schwarzengrund-03-19/index.html]. In 2007, *S*. Schwarzengrund outbreaks were also linked to dry pet food (Centers for Disease Control and Prevention, 2008).

This increase in the number of *S*. Schwarzengrund infections is not the only important factor to consider; there is also evidence of a higher frequency of antimicrobial resistance (AMR) within the strains of this serovar that is spreading internationally. Using antimicrobial susceptibility testing and pulsed field gel electrophoresis (PFGE) typing, Aarestrup et al. (2007) found that 7 of 14 strains isolated from humans in Denmark shared PFGE patterns with isolates from humans and chicken meat in Thailand, while 22 of 390 human-source isolates from the U.S. also had common profiles to those in Denmark and Thailand. These isolates showed a high frequency of resistance to nalidixic acid, along with a reduced susceptibility to ciprofloxacin (Aarestrup et al., 2007). The first reported instance of fluoroquinolone-resistance in *S.* Schwarzengrund in the U.S. came from a strain isolated from a patient who had traveled to the Philippines (Akiyama and Khan, 2012). In a study conducted on raw chicken samples from marketplaces in Taiwan, most of the *S.* Schwarzengrund strains demonstrated a ACSSuT resistance type [resistant to ampicillin, chloramphenicol, streptomycin, sulfamethoxazole, and tetracycline (Chen et al., 2010)]. Many of these antibiotics are commonly used in a variety of treatments for animal and human infections. Consequently, antimicrobial resistance further complicates the treatment of Salmonellosis caused by *S.* Schwarzengrund, which can lead to higher morbidity and mortality (Nair et al., 2018).

*Salmonella enterica* possesses a wide range of virulence factors that facilitate the establishment of successful infections in animal and human hosts (Ochman et al., 1996; van der Heijden and Finlay, 2012). The majority of these virulence factors are encoded in the chromosome; however, some are harbored by plasmids (Waters and Crosa, 1991; Ochman et al., 1996; van der Heijden and Finlay, 2012; Khajanchi et al., 2016, 2017; Khajanchi, 2022). A broad range of plasmid incompatibility groups (Inc) have been found in *Salmonella* serovars (Khajanchi et al., 2016, 2017; Khajanchi, 2022). Among them, IncFIB plasmids often possess functions associated with colicin production, the aerobactin siderophore and Sit iron acquisition systems, and persistence in intestinal epithelial cells (Khajanchi et al., 2016, 2017; Khajanchi, 2022).

Johnson et al. (2010) showed that horizontal gene transfer of IncFIB plasmids contributed to the acquisition of antimicrobial resistance. In their study, 902 *Salmonella* isolates that belonged to 59 different serovars were examined for plasmids. The IncFIB plasmids were found in isolates of serovars Kentucky, Typhimurium, and Heidelberg. It was shown that a single PFGE clonal type of *S.* Kentucky harbored these plasmids and the acquisition of the plasmid allowed *S*. Kentucky to be more competitive in colonizing the chicken cecum compared to those lacking the plasmid. Evaluation of sequences from three IncFIB plasmids from *S.* Kentucky isolates that originated in different locations at different times from different sources showed almost identical genetic sequence. These findings point to the IncFIB plasmid being recently attained within the *S.* Kentucky serovar., with a rapid transfer among the population that improved colonization and fitness abilities (Johnson et al., 2010). In other research on horizontal gene transfer, two IncFIB plasmids (pAPEC-O2-ColV and pAPEC-O2-R) were transferred into an avirulent and plasmid-less *E. coli* strain. The *E. coli* strain became virulent toward chick embryos and showed resistance to ampicillin, streptomycin, and several other antibiotics (Johnson et al., 2006).

IncFII and IncFIC plasmids contribute to the horizontal transfer of antimicrobial resistance genes including extended spectrum β-lactamases (ESBL; Yoon and Lee, 2022; de Jesus Bertani et al., 2023). Some IncFIC plasmids are fusion plasmids and carry both the IncFIC and IncFII replicons (Yoon and Lee, 2022). The formation and spread of fusion plasmids in *Enterobacteriaceae* is an emergent problem (Liu et al., 2021). Mobile genetic elements, such as insertion sequences and transposons can contribute to the formation of fusion plasmids (Liu et al., 2021). Therefore, to better understand the spread of fusion plasmids, it is important to characterize *Salmonella* and other enteric bacteria that harbor them. The objectives of the study were: (i) to perform molecular characterization of the IncFIB-IncFIC(FII) fusion plasmid of *S*. Schwarzengrund isolated from food and clinical sources; (ii) to determine the role of IncFIB-IncFIC(FII) fusion plasmid in invasion and persistence of *Salmonella*.

## Materials and methods

### Bacterial strains

A total of 55 *S.* Schwarzengrund isolates, of which 36 were collected from human patients, were obtained from the Wisconsin State Lab of Hygiene (WLSH; *n* = 15), Minnesota Department of Health (MDH; *n* = 18) and the Maryland Department of Health (MD; *n* = 3); while 19 food isolates were collected as part of the National Antimicrobial Resistance Monitoring System (NARMS) efforts. Food isolates were primarily collected from chicken, while clinical isolates were collected from stool, urine, and blood (Supplementary Table 1). The sodium azide resistant *Escherichia coli* J53 was used as a recipient for the conjugation studies (Jacoby and Han, 1996).

### Whole genome sequencing using short read and long read methods

Of these 55 isolates, 17 fusion plasmid-containing *S*. Schwarzengrund isolates were sequenced using both short read Illumina and long-read Oxford Nanopore Technology (ONT; Sopovski et al., 2024). The remaining 38 isolates were only sequenced using the short read Illumina platform. CheckM was used to determine the completeness and contamination of the short read assemblies. Quality measurements of raw data and assembled genomes were previously published separately (Khajanchi et al., 2019a; Sopovski et al., 2024) and also provided as Supplementary Table 2.

Short-read WGS was performed by a procedure described earlier (Khajanchi et al., 2016). Briefly, genomic DNA from bacterial cells was extracted using a DNeasy Blood and Tissue kit (Qiagen, Valencia, CA, United States). The quality and quantity of the DNA were examined by Nanodrop (ThermoFisher Scientific, Grand Island, NY, United States) and the Qubit BR assay kit (ThermoFisher Scientific). DNA libraries were constructed using 1 ng of DNA from each sample using the Nextera XT DNA library preparation kit (Illumina, San Diego, CA, United States). Samples were multiplexed using combinations of two indexes of Nextera XT Index Kit. DNA samples were diluted, denatured, loaded and sequenced on an Illumina MiSeq instrument with 2 × 250 pair-end chemistry.

For long-read ONT sequencing, the 1D native barcoding genomic DNA long read selection protocol was used with the SQK-LSK109 kit (Oxford Nanopore, Oxford, UK) as described earlier (Taylor et al., 2019). Briefly, 1 μg of DNA was subjected to end repair and dA-tailing using the NEBNext® Ultra™ II End Repair/dA-Tailing module (New England Biolabs, Ipswich, MA, United States). End-prepped DNA fragments were barcoded using the EXP-NBD104 and EXP-NBD114 kits (Oxford Nanopore). Equimolar amounts of each barcoded sample were pooled together, adapters were ligated, and the resulting library pools were sequenced on a MinION device using a FLO-MIN106 (R9.4.1) flow cell for 48 h.

The nanopore reads were trimmed and filtered using NanoFilt (v2.3.0; De Coster et al., 2018). For NanoFilt, the parameters were set to filter out nanopore reads with a quality score (qscore) of less than 10 or if the read was less than 500 bp long. The short and long-read sequences were assembled by hybrid assembly using UniCycler (v0.4.8; Wick et al., 2017). The FASTA files of the assemblies from each isolate were analyzed using PlasmidFinder (version 2.1) and ResFinder (version 4.1) to identify predicted plasmids and antimicrobial resistance genes, respectively (Supplementary Table 1; Zankari et al., 2012; Carattoli et al., 2014).

### Single nucleotide polymorphism analysis

WGS-based SNP analysis of the 55 *Salmonella* genomes was performed using the FDA Center for Food Safety and Applied Nutrition (CFSAN) SNP pipeline (Davis et al., 2015). The CFSAN SNP pipeline was used to find the pairwise SNP distances between the isolates. The hybrid assembly for isolate WLSH7 was used as reference for the SNP analysis. FastTree (v2.1.11), using the general time reversible model, was used to approximate the maximum likelihood phylogeny with 1,000 bootstraps. The phylogeny was visualized in FigTree (v1.4.4).

### Plasmid annotation and phylogeny

Platon (v1.6) was used to annotate the assembly contigs for plasmids. NCBI’s online BLAST server was used to identify the best hits (≥99% identity and ≥ 70% query coverage) in the NCBI Nucleotide database to the IncFIB-IncFIC(FII) plasmid, which were downloaded. Genes were annotated in the plasmids using Prokka (v1.14.5). The pangenome was estimated with Roary (v3.12.0). For reference, the pangenome contained 631 genes of which 49 were core. MAFFT (v7.305) was used to align the concatenated 49 core genes. FastTree (v2.1.11), using the general time reversible model, was used to approximate the maximum likelihood phylogeny with 1,000 bootstraps. The phylogeny was visualized in FigTree (v1.4.4). The plasmid annotations were visualized in SnapGene (SnapGene by Dotmatics, Boston, MA, United States). The tanglegram was created using R and the cophyloplot function from the ape package.

### Bacterial conjugation

Conjugation experiments were carried out to determine the transferability of IncFIB plasmids in *S.* Schwarzengrund isolates either by plate mating or broth mating approaches (Khajanchi et al., 2019b). In plate mating strategy, IncFIB-IncFIC(FII) positive *S*. Schwarzengrund isolates (donors) and the sodium azide resistant recipient *E. coli* J53 were cross streaked on Luria-Bertani (LB; BD, Franklin Lakes, NJ, United States) agar plates. After 24 h of incubation, the cells from the intersection were collected and re-streaked onto selective plates containing sodium azide (350 μg/mL) and streptomycin (16 μg/mL). Individual colonies were picked and sub-cultured onto MacConkey agar to confirm *E. coli* colonies. Carriage of IncFIB-IncFIC(FII) plasmids by *E. coli* transconjugants were confirmed by PCR. Conjugation experiments that were unsuccessful by plate mating were subjected to a different approach described in our previous study (Khajanchi et al., 2019b). Briefly, a single colony of IncFIB-IncFIC(FII) containing *S.* Schwarzengrund isolates (donor) and *E. coli* J53 (recipient) was grown separately in LB broth overnight at 37°C. The recipient and the donor were subsequently mixed together in a 1:1 proportion and centrifuged at 7,000 × g for 5 min to obtain the pellet. The pellets were dispersed in 250 μL of LB broth and spotted onto a LB agar plate. The plate was incubated for 3–4 h at 37°C in upright position. The growth seen was suspended in 1 mL phosphate buffered saline (PBS) and 100 μL of cell suspension was spread onto a LB agar plate containing sodium azide (350 μg/mL) and streptomycin (16 μg/mL). Pink colonies were selected after streaking on to selective MacConkey agar plate and presence of IncFIB-IncFIC(FII) was confirmed by PCR.

### Virulome and plasmid transfer gene assay

The detection of virulence and plasmid transfer-associated genes encoded by the *Salmonella* isolates were predicted based on their whole genome sequences. Genome sequences from *Salmonella* donors and transconjugants (plasmids in *E. coli* J53) were trimmed, and *de novo* assembly was completed using CLC Genomics Workbench (ver. 9.0, Qiagen, Redwood City, CA, United States). FASTA files of sequence assemblies from each strain were analyzed using the multiple sequence Comparison tool within the FDA Virulence and AMR Plasmid Transfer Factor Database,^1^ which targets 594 putative *Salmonella* virulence genes and plasmid transfer genes from key AMR plasmid Inc. groups (Aljahdali et al., 2020; Tate et al., 2022; Algarni et al., 2023). The predicted presence and absence data for the putative virulence and plasmid transfer genes were downloaded from the database, transformed to binary data and imported into BioNumerics for phylogenetic analyses of the virulence and plasmid transfer genes. Based categorical (binary) difference calculations and dendrograms were generated using UPGMA (Applied Maths, Austin, TX, United States). The profiles of the presence/absence of virulence and plasmid transfer genes were further compared in BioNumerics using minimum spanning tree analyses to compare similarities of the wildtype and transconjugant strains.

### Bacterial invasion assay

Bacterial invasion assays were performed using human intestinal epithelial cells (Caco-2) as described previously (Khajanchi et al., 2017). Briefly, 10^5^ Caco-2 cells per well were seeded in 24-well tissue culture plates and incubated at 37°C overnight in a 5% CO_2_ incubator. Cells in one of the wells were counted using a Cellometer Auto T4 (Nexcelom Bioscience, Lawrence, MA, United States) and the Caco-2 cells were infected with different *S.* Schwarzengrund isolates at multiplicity of infection (MOI) of 10. After incubation for 1 h at 37°C, the cells were washed twice with PBS to remove bacteria that had not infected the Caco-2 cells and incubated with 200 μg/mL of gentamicin. After incubation for 1 h at 37°C, the cells were washed twice with PBS and lysed with 0.1% chilled Triton X-100, followed by dilution and plating on trypticase soy agar (TSA) to obtain colony forming unit counts (CFUs) of bacteria following overnight incubation at 37°C. Three replicates per strain were included and the experiments were repeated three times.

### Bacterial persistence assay

Caco-2 cells were infected as for the invasion assay. After 1 h incubation, the cells were washed twice with PBS and incubated with 100 μg/mL of gentamicin for 48 h. After incubation, cells were washed, lysed, and CFUs were counted as in the invasion assay procedure, with three replicates per strain and experiments carried out in triplicate.

## Results

### SNP analyses

**Figure 1:**
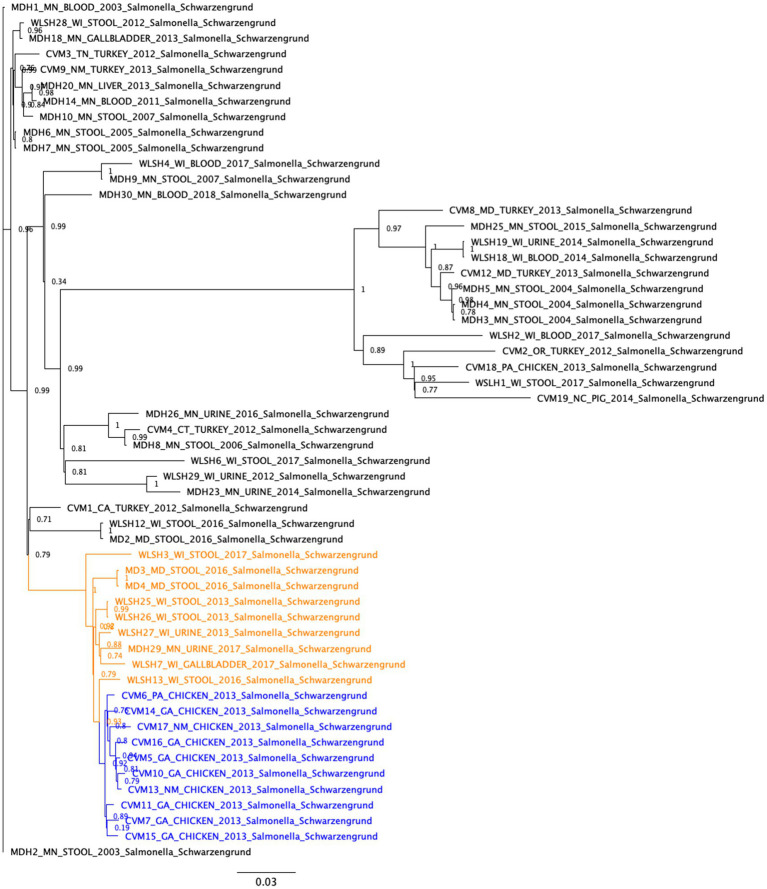
Single nucleotide polymorphism (SNP)-based phylogenetic analyses of *Salmonella* Schwarzengrund isolates from food and clinical sources. The SNP differences were identified with the CFSAN SNP Pipeline and the phylogeny was created with FastTree using the general time reversible model and 1,000 bootstraps. The isolates carrying the IncFIB-IncFIC(FII) plasmid formed a closely related cluster (between 0 and 52 SNP differences). These isolates further separated into subclusters by human and chicken isolation sources. The clinical and food isolates carrying the fusion plasmid are highlighted in orange and blue, respectively.

SNP analysis (Figure 1) showed that the 17 food and clinical isolates carrying the IncFIB-IncFIC(FII) fusion plasmid clustered within the same subclade (between 0 and 52 SNP differences), separated from the other isolates that lacked the fusion plasmids (Figure 1). This suggests the plasmid was acquired and has been maintained by that subclade lineage. The two exceptions were isolates CVM-6 and WLSH-27 which lacked the fusion plasmid but still clustered with the isolates carrying the fusion plasmid. These two isolates carried an IncFIC plasmid that was near-identical to the IncFIB-IncFIC(FII) fusion plasmid over ~63% of its length, indicating it had not undergone fusion or had lost the IncFIB portion of the fusion plasmid. Within the fusion plasmid subclade, isolates further separated into subclusters by human and chicken isolation sources. However, this difference might be an artifact due to the time of collection because all the chicken isolates were collected in 2013, whereas the clinical isolates were collected between 2013 and 2017.

### The concatenated core gene phylogeny of the IncFIB-IncFIC(FII) fusion plasmid

**Figure 2:**
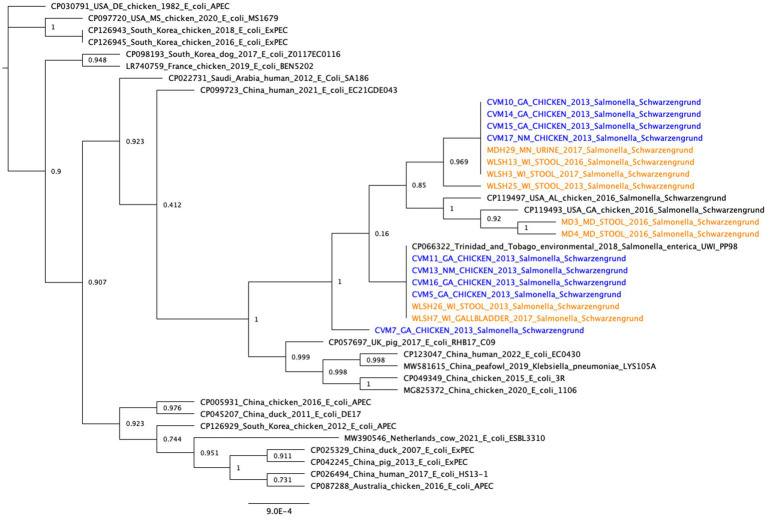
Core gene phylogeny of the IncFIB-IncFIC(FII) plasmid. Includes the plasmids from the 17 long-read sequenced isolates and the best BLAST hits from the NCBI Nucleotide database (≥99% identity and ≥70% query coverage). Clinical isolates are in orange and food isolates are in blue. The pangenome of all the plasmids (including the 17 isolates from this study and the best BLAST hits in NCBI) had 631 genes, of which 49 were core. The median number of genes per plasmid sequence was 158.

**Figure 3:**
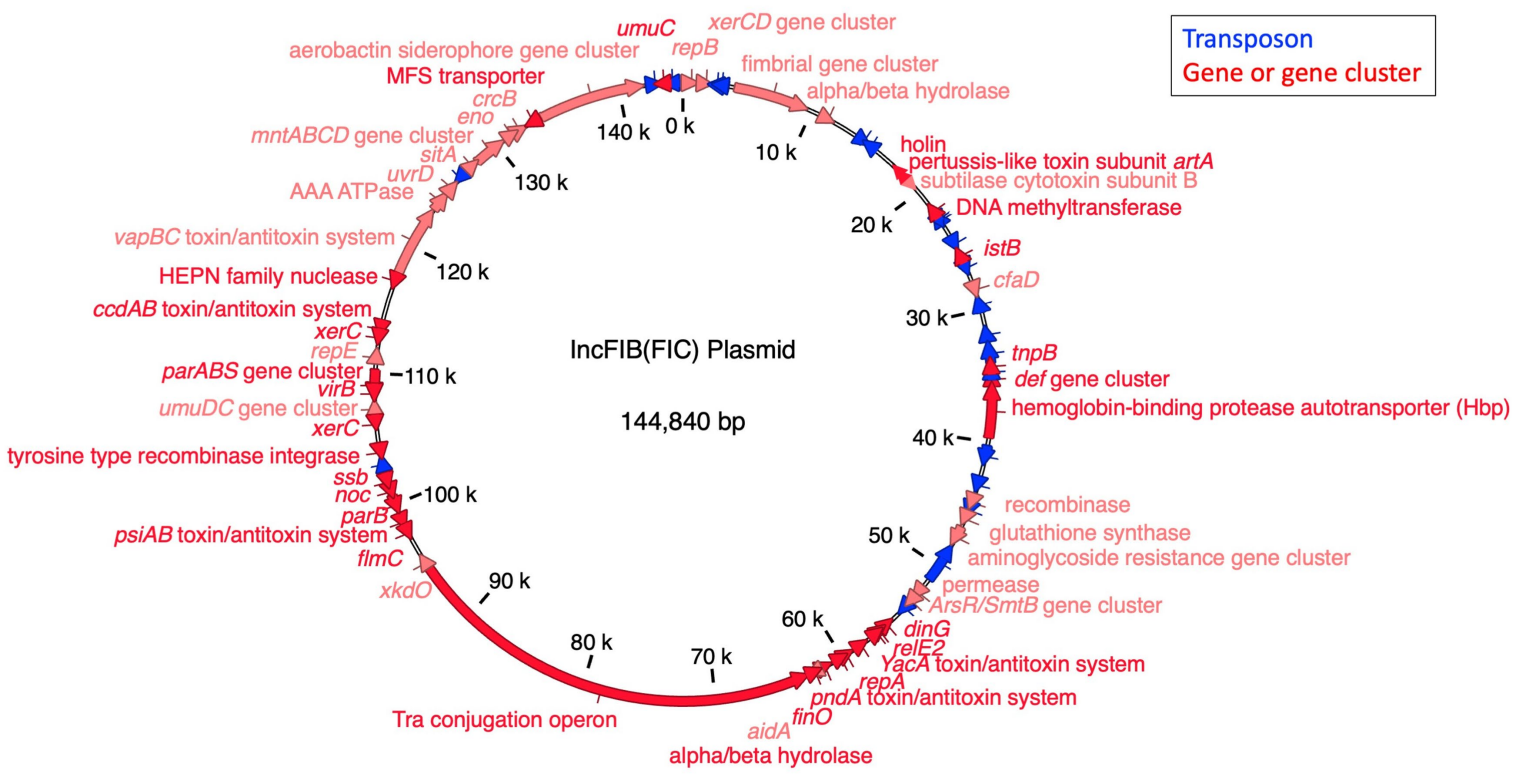
The annotated IncFIB-IncFIC(FII) plasmid. The markers for the FIB and FIC replicons co-occurred in the plasmid from 1–344 bp to 59,325–59,519 bp, respectively, indicating it is a fusion plasmid.

The topology of the IncFIB-IncFIC(FII) fusion plasmid phylogeny differed from the SNP tree in Figure 1, with no clear separation between the human and chicken isolates (Figure 2). Among the best BLAST hits in the NCBI Nucleotide database were plasmids from isolates that were recovered from animals (e.g., chicken, duck, pig, peafowl) with colibacillosis and respiratory disease. Further, several related plasmids were isolated from avian pathogenic *E. coli* (APEC). The gene annotation of the IncFIB-IncFIC(FII) plasmid can be seen in Figure 3. The pangenome of the IncFIB-IncFIC(FII) plasmid contained 631 genes of which 49 were core.

### Bacterial conjugation

Among 19 food isolates, nine contained the IncFIB-IncFIC(FII) fusion plasmid (CVM-5, CVM-7, CVM-10, CVM-11, CVM-13, CVM-14, CVM-15, CVM-16, CVM-17; Supplementary Table 1). All nine isolates successfully conjugated with *E. coli* J53. Of the 36 clinical isolates, eight isolates contained the IncFIB-IncFIC(FII) fusion plasmid (MDH29, WLSH-3, WLSH-7, WLSH-13, WLSH-25, WLSH-26, MD-3, MD-4; Supplementary Table 1). Out of the eight isolates, three (WLSH 7, WLSH 13, WLSH 25) successfully conjugated with *E. coli* J53. The conjugation experiment was repeated for the five isolates that did not transfer the plasmid, but conjugation still did not occur, indicating they might not be conjugative under the test conditions. When the sequenced plasmids in the wild type and transconjugant isolates (J53::CVM5, J53::CVM7, J53::CVM10, J53::CVM11, J53::CVM13, J53::CVM14, J53::CVM15, J53::CVM16, J53::CVM17, J53::WLSH-7, J53::WLSH-13, J53::WLSH-25) were compared, they had the same gene content (Table 1), including antimicrobial resistance and iron acquisition genes, supporting the successful conjugation of the fusion plasmid.

**Table 1:** Sequence analyses of wild type and transcongugants of 12 IncFIB containing *Salmonella* Schwarzengrund food and clinical isolates.

Wild type isolates				Transconjugants generated using *Escherichia coli* J53 as recipient			
Wildtype Strain ID	Palsmid content	Resistance gene	Iron acquisition genes	Transconjugants ID	Plasmid content	Resistance gene	Iron acquisition genes
CVM −5	IncFIB(AP001918), IncFIC(FII)	*aac(6′)-Iaa, aph(3″)-Ib, aph(6)-Id*	*iucABCD/iutA/iroNB/sitABCD*	J53::CVM-5	IncFIB(AP001918), IncFIC(FII)	*aph(3″)-Ib, aph(6)-Id*	*iucABCD/iutA/sitABCD*
CVM-7	IncFIB(AP001918), IncFIC(FII)	*aac(6′)-Iaa, aph(3″)-Ib, aph(6)-Id*	*iucABCD/iutA/iroNB/sitABCD*	J53::CVM-7	IncFIB(AP001918), IncFIC(FII)	*aph(3″)-Ib, aph(6)-Id*	*iucABCD/iutA/sitABCD*
CVM-10	IncFIB(AP001918), IncFIC(FII)	*aac(6′)-Iaa, aph(3″)-Ib, aph(6)-Id*	*iucABCD/iutA/iroNB/sitABCD*	J53::CVM-10	IncFIB(AP001918), IncFIC(FII)	*aph(3″)-Ib, aph(6)-Id*	*iucABCD/iutA/sitABCD*
CVM-11	IncFIB(AP001918), IncFIC(FII)	*aac(6′)-Iaa, aph(3″)-Ib, aph(6)-Id*	*iucABCD/iutA/iroNB/sitABCD*	J53::CVM-11	IncFIB(AP001918), IncFIC(FII)	*aph(3″)-Ib, aph(6)-Id*	*iucABCD/iutA/sitABCD*
CVM-13	IncFIB(AP001918), IncFIC(FII)	*aac(6′)-Iaa, aph(3″)-Ib, aph(6)-Id*	*iucABCD/iutA/iroNB/sitABCD*	J53::CVM-13	IncFIB(AP001918), IncFIC(FII)	*aph(3″)-Ib, aph(6)-Id*	*iucABCD/iutA/sitABCD*
CVM-14	IncFIB(AP001918), IncFIC(FII)	*aac(6′)-Iaa, aph(3″)-Ib, aph(6)-Id*	*iucABCD/iutA/iroNB/sitABCD*	J53::CVM-14	IncFIB(AP001918), IncFIC(FII)	*aph(3″)-Ib, aph(6)-Id*	*iucABCD/iutA/sitABCD*
CVM-15	IncFIB(AP001918), IncFIC(FII)	*aac(6′)-Iaa, aph(3″)-Ib, aph(6)-Id*	*iucABCD/iutA/iroNB/sitABCD*	J53::CVM-15	IncFIB(AP001918), IncFIC(FII)	*aph(3″)-Ib, aph(6)-Id*	*iucABCD/iutA/sitABCD*
CVM-16	IncFIB(AP001918), IncFIC(FII)	*aac(6′)-Iaa, aph(3″)-Ib, aph(6)-Id*	*iucABCD/iutA/iroNB/sitABCD*	J53::CVM-16	IncFIB(AP001918), IncFIC(FII)	*aph(3″)-Ib, aph(6)-Id*	*iucABCD/iutA/sitABCD*
CVM-17	IncFIB(AP001918), IncFIC(FII)	*aac(6′)-Iaa, aph(3″)-Ib, aph(6)-Id*	*iucABCD/iutA/iroNB/sitABCD*	J53::CVM-17	IncFIB(AP001918), IncFIC(FII)	*aph(3″)-Ib, aph(6)-Id*	*iucABCD/iutA/sitABCD*
WLSH-7	IncFIB(AP001918), IncFIC(FII)	*aac(6′)-Iaa, aph(3″)-Ib, aph(6)-Id*	*iucABCD/iutA/iroNB/sitABCD*	J53::WLSH-7	IncFIB(AP001918), IncFIC(FII)	*aph(3″)-Ib, aph(6)-Id*	*iucABCD/iutA/sitABCD*
WLSH-13	IncFIB(AP001918), IncFIC(FII)	*aac(6′)-Iaa, aph(3″)-Ib, aph(6)-Id*	*iucABCD/iutA/iroNB/sitABCD*	J53::WLSH-13	IncFIB(AP001918), IncFIC(FII)	*aph(3″)-Ib, aph(6)-Id*	*iucABCD/iutA/sitABCD*
WLSH-25	IncFIB(AP001918), IncFIC(FII)	*aac(6′)-Iaa, aph(3″)-Ib, aph(6)-Id*	*iucABCD/iutA/iroNB/sitABCD*	J53::WLSH-25	IncFIB(AP001918), IncFIC(FII)	*aph(3″)-Ib, aph(6)-Id*	*iucABCD/iutA/sitABCD*

### Virulome and plasmid transfer gene analyses

**Figure 4:**
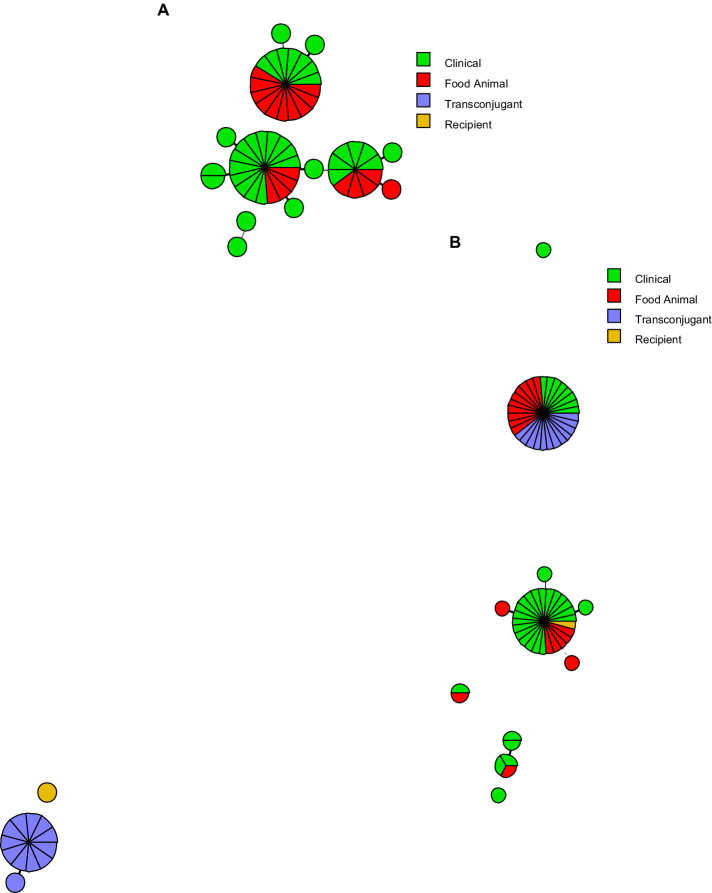
Minimum spanning tree analyses of the **(A)** *Salmonella* virulence factors and **(B)** the plasmid transfer genes. The colors correspond to the sample types and the size of the circles are representative of the number of strains sharing common virulence or transfer gene profiles. The distance between the circles is representative of the number of genes differences in the different groups. The closer the circles the more similar the gene profiles. In panel **(A)**, the recipient and transconjugants are *E. coli* strains and as such lack many of the *Salmonella* virulence factors and are distinct from the *Salmonella* isolates. In Panel **(B)**, those grouped closest the letter B are the strains that carry the IncFIB-IncFIC(FII) plasmid.

The presence of the *Salmonella*-associated virulence genes are detailed in Supplementary Table 3 and the comparison of the virulence gene profiles are shown in Supplementary Figure 1. Overall, the *S*. Schwarzengrund isolates had very similar virulence factor profiles, with the exception of putative virulence genes associated with the IncFIB-IncFIC(FII) plasmids, including *iucABCD* and *iutA* of the aerobactin operon and *traT*. The transconjugants separated to a distinct clade, as they were *E. coli* and lacking many of the *Salmonella*-associated virulence genes (Supplementary Figure 2). When the plasmid transfer genes were detected, the most common genes detected were the IncFIB-IncFIC(FII)-associated genes that were present in the transconjugants and their corresponding donor strains (top clade in Supplementary Figure 2). One of the donor strains (WLSH-3) carried the IncFIB-IncFIC(FII) along with IncI1 and IncI2-associated transfer genes. A group of five other strains carried IncI1 plasmid-associated genes, without the IncFIB-IncFIC(FII) plasmids (bottom group of Supplementary Figure 2), and two strains carried an IncHI2 plasmid. These results correlated with the plasmid replicon typing results (Supplementary Table 1). The relatedness of the virulence genes (Figure 4A) and plasmid transfer genes (Figure 4B) can also be displayed with the minimal spanning trees. The three largest groups of *Salmonella* differ by the presence of the aerobactin/*traT* genes (top group) and the absence of *sopE* and *Salmonella* genomic island (SGI)-1-associated insertion genes (*int* and *xis;* group to the right central group). The majority of the SGI-1 genes were absent in all of the strains in the study. The plasmid groups are largely separated by those with the IncFIB-IncFIC(FII) plasmid (top half of Figure 4B) and those lacking the plasmid (bottom groups).

### Invasion and persistence assay

**Figure 5:**
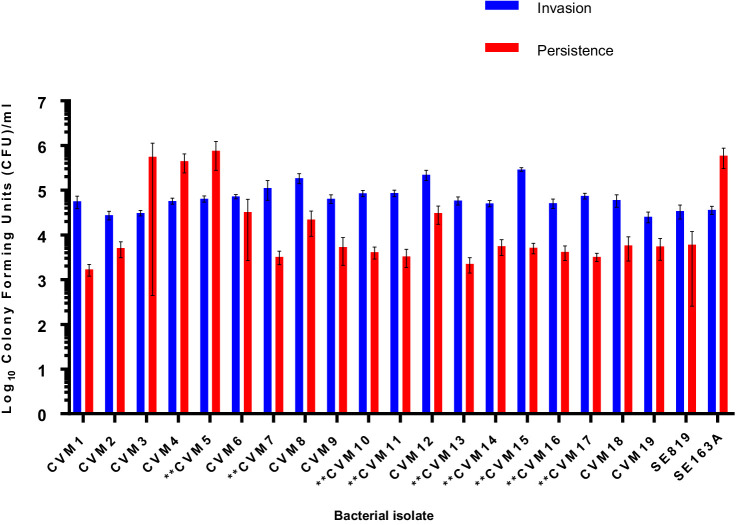
Invasion and persistence of 19 wildtype *Salmonella* Schwarzengrund food isolates. Nine food isolates contained IncFIB-IncFIC(FII)are indicated by asterisks. The general trend was that the amount of surviving colony forming units were lower after 48 h (persistence) as compared to 1 h (Invasion). X-axis indicates the number of isolates (CVM 1 to CVM-19), SE819 [less virulent strain that lacked IncFIB-IncFIC(FII)], SE163A (virulent strain that contained IncFIB along with other virulence associated plasmids). Blue bars indicate invasion and red bars indicate persistence. Error bars indicate standard error of mean. The difference between invasion and persistence were analyzed by Student t-test (two-tailed). A *p* ≤ 0.05 was considered significantly different between two groups. The difference between invasion and persistence rate for food isolates was statistically significant (*p* = 0.007).

**Figure 6:**
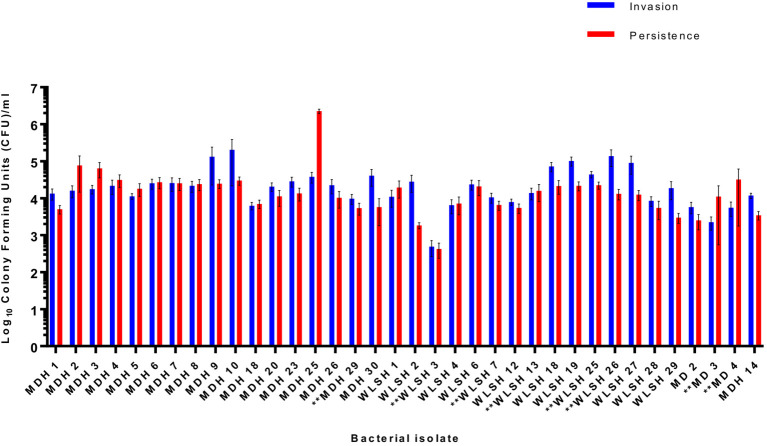
Invasion and persistence of 36 wildtype *Salmonella* Schwarzengrund clinical isolates. Eight clinical isolates containing IncFIB-IncFIC(FII) are indicated by asterisks. The general trend was that the number of surviving colony-forming units was lower in persistence as compared to invasion. X-axis indicates the number of isolates with sample ID and Y-axis represents the CFU/ml. Blue bars indicate invasion and red bars indicate persistence. Error bars indicate standard error of mean. The difference between invasion and persistence were analyzed by Student t-test (two-tailed). A *p* ≤ 0.05 was considered significantly different between two groups. The difference between invasion and persistence rate for clinical isolates was statistically non-significant (*p* = 0.1192).

To assess the role of the IncFIB-IncFIC(FII) plasmid in invasion and persistence in host cells, human Caco-2 cells were infected with *S.* Schwarzengrund food and clinical isolates (Figure 5). The general trend, for both food and clinical isolates, was that the amount of surviving CFUs of fusion plasmid containing strains were lower after 48 h (persistence) as compared to 1 h (Invasion). The difference between invasion and persistence rate was statistically significant for food isolates (*p* = 0.007), however; this difference was statistically non-significant for clinical isolates (*p* = 0.1192). We observed that IncFIB-IncFIC(FII) plasmid did not significantly add to the invasion and persistence potential of isolates when compared to those without the plasmids (Figures 5,6).

## Discussion

IncFIB plasmids can contain both antimicrobial resistance genes and a wide range of virulence factors, hence dissemination of these plasmids in food pathogens is a public health concern. IncFIB and IncFIB-like plasmids are more commonly harbored by *Salmonella* and APEC, which could serve as reservoir for the spread of these plasmids into other Gram-negative bacteria (Johnson et al., 2006, 2010; Khajanchi et al., 2017). In this study, we extensively characterized *S*. Schwarzengrund isolated from food and clinical samples, some of which contained IncFIB-IncFIC(FII) fusion plasmids. In the strains isolated from food samples, all the IncFIB-IncFIC(FII) plasmid containing *S*. Schwarzengrund strains were isolated from chicken sources, indicating that IncFIB-IncFIC(FII) plasmids may be associated with host specific advantages. In clinical isolates, the majority of the IncFIB-IncFIC(FII) plasmid containing *S*. Schwarzengrund strains were isolated from stool and two strains were isolated from urine and the gallbladder. These data suggested that IncFIB-IncFIC(FII) plasmids might play a role in extra-intestinal *Salmonella* infection; however, further study is warranted to provide an experimental basis for this speculation. In addition, acquisition of a similar plasmid such as ColV plasmid by *S.* Kentucky enabled its extraintestinal virulence in chickens (Johnson et al., 2010). It is interesting to note that isolates carrying the IncFIB-IncFIC(FII) fusion plasmid formed a subclade in the SNP phylogeny, separated from the isolates lacking the fusion plasmid. This result implies that the isolates with IncFIB-IncFIC(FII) plasmid is a lineage defined by the acquisition of the IncFIB-IncFIC(FII) plasmid. These data agreed with our previous findings on *S. Typhimurium* in which IncFIB containing *S. Typhimurium* isolates were clustered together and separated from the isolates that did not carry IncFIB plasmids (Khajanchi et al., 2017). Additionally, these findings indicate that there is a possible epidemiological link between IncFIB-IncFIC(FII) containing food and clinical isolates, which warrants further investigation. Additionally, out of eight IncFIB-IncFIC(FII) plasmid containing isolates from humans, two patients reported eating undercooked chicken; while the sources of the remaining six isolates were unknown, with patients either not remembering their exposures to food-related sources or did not agree to an interview.

A tanglegram showed the lack of congruence between the whole genome SNP analysis and the plasmid phylogenies (Supplementary Figure 3). The different topologies of the fusion plasmid carrying isolates in the whole genome phylogeny (Figure 1) and the concatenated core gene plasmid phylogeny (Figure 2) indicates that, although the lineage might have acquired and maintained the plasmid, the plasmid still might have been horizontally transferred within the lineage.

The association of the best NCBI BLAST hit plasmids with human and animal illness supports a possible association with virulence. The virulome analyses showed a similar virulence and plasmid profile for food and clinical isolates. These commonalities are quite evident in Figure 4, where each of the *Salmonella* groupings, for both virulence genes and plasmid genes, have a least three isolates from both food animal and clinical sources. In the virulome analyses, there was some separation of the isolate cluster carrying the IncFIB-IncFIC(FII) fusion plasmids vs. the isolates that lack the fusion plasmid. These differences were due to virulence-associated genes carried on the plasmids (aerobactin operon and *traT*, primarily). Among the isolates not carrying the fusion plasmid, there were two larger subgroups that differed in the carriage of *sopE, int*, and *xis*. The two latter genes are associated with SGI-1. Most of the other SGI-1 associated genes in the database, with the exceptions of *res* and *yidY,* were absent in all of the isolates.

The conjugation of IncFIB and IncFIB-like plasmids has been linked to enhanced virulence in the recipient bacteria. For example, avirulent avian *E. coli* in chick embryo models became virulent after acquiring IncFIB plasmids through transformation (Wooley et al., 1996) and IncFIB transconjugants had increased colonization survival in host compared to recipients (Khajanchi et al., 2017). The *S.* Schwarzengrund IncFIB-IncFIC(FII) fusion plasmids were conjugative as evidenced by their transferability to *E. coli*. The only exception were five clinical isolates whose fusion plasmid did not transfer to *E. coli*, after several attempts. The genetic cause of this could not be determined. Studies suggest that the spread of IncFIB plasmids may lead to improved survival in humans and food animals, increasing the chance for human infection (Johnson et al., 2010; Khajanchi et al., 2017). The spread of antimicrobial resistance genes has also been linked to IncFIB plasmids (Han et al., 2012). Dissemination of IncFIB plasmids along with antimicrobial resistance genes may decrease the effectiveness of antimicrobial therapies for diseases (Khajanchi et al., 2017).

As noted in previous studies, IncFIB plasmids possess several factors that enhance bacterial pathogenicity, such as the production of colicins that kill closely related species, the immunity gene that protects the bacteria from its own bacteriocin, plasmid transfer genes, and the aerobactin system that allows bacteria to combat the host’s immunity and to sequester iron for its own survival (Weinberg, 1978; Waters and Crosa, 1991; Khajanchi et al., 2017; Khajanchi, 2022). Various research has been conducted on the contribution of IncFIB plasmids on microbial pathogenesis. However, more work is needed to answer questions about host range, host specificity, environmental sources, and the role of virulence factors encoded by the IncFIB and similar plasmids in bacterial pathogenicity. One aim of this study was to evaluate the virulence capacity of IncFIB-IncFIC(FII) containing *S*. Schwarzengrund by conducting invasion and persistence assessments using tissue culture assay on Caco-2 cells. Previous studies have shown that IncFIB plasmids increased the colonization of chickens by *E. coli* (Ginns et al., 2000), and also enhanced the ability of *S*. Kentucky to colonize chickens (Johnson et al., 2010). Our previous study showed that IncFIB transconjugants had higher invasion and persistence, suggesting that IncFIB plasmids can increase colonization of pathogens in the gut (Khajanchi et al., 2017).

In the present study, Figures 5, 6 show that the IncFIB-IncFIC(FII) plasmids did not significantly contribute to the invasion or persistence of *S*. Schwarzengrund strains isolated from food and clinical sources. The contribution of IncFIB encoded virulence factors in *Salmonella* pathogenesis is still not completely understood. IncFIB or similar plasmids could be diverse in nature and may play distinct roles in different *Salmonella* serovars.

## Conclusion

The core gene phylogeny of the IncFIB-IncFIC(FII) fusion plasmid revealed that the *S*. Schwarzengrund isolates might be descended from the plasmids of avian pathogenic isolates, indicating it might confer adaptation to avian hosts. Further, the fusion plasmid carried several virulence factors that might increase the pathogenicity of its bacterial host. This study shows that the IncFIB-IncFIC(FII) fusion plasmids can be transferred between Enterobacteriaceae species. Though the plasmid has virulence factors such as iron acquisition systems; toxin-antitoxin modules that should increase the pathogenicity of *Salmonella*, our assays showed that there was no difference in invasion and persistence for the isolates with or without of IncFIB-IncFIC(FII). More research is needed to determine the correlation between the virulence factors and the overall pathogenicity of *S*. Schwarzengrund. Future studies will explore the invasion and persistence of *Salmonella* transconjugants in tissue culture. This study highlights that a better understanding of the role of plasmids in *Salmonella* pathogenesis is needed, and that plasmids might be a significant microbiological hazard associated with food.

## Data availability statement

The datasets presented in this study can be found in online repositories. The names of the repository/repositories and accession number(s) can be found in the article/Supplementary material.

## Ethics statement

The studies involving humans were approved by Minnesota Department of Health, Maryland Department of Health, Wisconsin State Lab of Hygiene. The studies were conducted in accordance with the local legislation and institutional requirements. The participants provided their written informed consent to participate in this study.

## Author contributions

MF: Investigation, Methodology, Writing – original draft. DS: Data curation, Investigation, Writing – original draft. SC: Methodology, Writing – review & editing, Data curation. NY: Methodology, Writing – review & editing, Investigation. NA: Investigation, Methodology, Writing – review & editing. CG: Investigation, Methodology, Writing – review & editing. CA: Investigation, Methodology, Writing – review & editing. AC: Investigation, Methodology, Writing – review & editing. JH: Investigation, Methodology, Writing – review & editing. YS: Investigation, Methodology, Writing – review & editing. SZ: Investigation, Methodology, Writing – review & editing, Resources. XW: Methodology, Resources, Writing – review & editing. SF: Methodology, Resources, Writing – review & editing, Data curation, Funding acquisition, Investigation. BK: Investigation, Methodology, Writing – review & editing, Conceptualization, Supervision, Writing – original draft.

## Footnotes

^1^ https://virulence.fda.gov

## References

[ref1] Aarestrup F. M. Hendriksen R. S. Lockett J. Gay K. Teates K. McDermott P. F.. (2007). International spread of multidrug-resistant *Salmonella* Schwarzengrund in food products. Emerg. Infect. Dis. 13, 726–731. doi: 10.3201/eid1305.061489, PMID: 17553251 PMC2738437

[ref2] Akiyama T. Khan A. A. (2012). Molecular characterization of strains of fluoroquinolone-resistant *Salmonella enterica* serovar Schwarzengrund carrying multidrug resistance isolated from imported foods. J. Antimicrob. Chemother. 67, 101–110. doi: 10.1093/jac/dkr414, PMID: 22010209

[ref3] Algarni S. Foley S. L. Tang H. Zhao S. Gudeta D. D. Khajanchi B. K.. (2023). Development of an antimicrobial resistance plasmid transfer gene database for enteric bacteria. Front Bioinform 3:1279359. doi: 10.3389/fbinf.2023.1279359 38033626 PMC10682676

[ref4] Aljahdali N. H. Khajanchi B. K. Weston K. Deck J. Cox J. Singh R.. (2020). Genotypic and phenotypic characterization of incompatibility group FIB positive *Salmonella enterica* Serovar typhimurium isolates from food animal sources. Genes 11:1307. doi: 10.3390/genes11111307, PMID: 33158112 PMC7716204

[ref5] Andino A. Hanning I. (2015). *Salmonella enterica*: survival, colonization, and virulence differences among serovars. ScientificWorldJournal 2015:520179, 1–16. doi: 10.1155/2015/520179 25664339 PMC4310208

[ref6] Asai T. Murakami K. Ozawa M. Koike R. Ishikawa H. (2009). Relationships between multidrug-resistant *Salmonella enterica* Serovar Schwarzengrund and both broiler chickens and retail chicken meats in Japan. Jpn. J. Infect. Dis. 62, 198–200. doi: 10.7883/yoken.JJID.2009.198, PMID: 19468180

[ref7] Bangtrakulnonth A. Pornreongwong S. Pulsrikarn C. Sawanpanyalert P. Hendriksen R. S. (2004). Lo Fo Wong DM, Aarestrup FM: *Salmonella* serovars from humans and other sources in Thailand, 1993-2002. Emerg. Infect. Dis. 10, 131–136. doi: 10.3201/eid1001.02-0781, PMID: 15078609 PMC3322742

[ref8] Carattoli A. Zankari E. Garcia-Fernandez A. Voldby Larsen M. Lund O. Villa L.. (2014). In silico detection and typing of plasmids using PlasmidFinder and plasmid multilocus sequence typing. Antimicrob. Agents Chemother. 58, 3895–3903. doi: 10.1128/AAC.02412-14, PMID: 24777092 PMC4068535

[ref9] Centers for Disease Control and Prevention (2008). Update: recall of dry dog and cat food products associated with human *Salmonella* Schwarzengrund infections--United States. MMWR Morb. Mortal. Wkly Rep. 57, 1200–1202, 18987615

[ref10] Chen M. H. Wang S. W. Hwang W. Z. Tsai S. J. Hsih Y. C. Chiou C. S.. (2010). Contamination of *Salmonella* Schwarzengrund cells in chicken meat from traditional marketplaces in Taiwan and comparison of their antibiograms with those of the human isolates. Poult. Sci. 89, 359–365. doi: 10.3382/ps.2009-00001, PMID: 20075291

[ref11] Davis S. Pettengill J. Lou Y. Payne J. Shpuntoff A. Rand H.. (2015). CFSAN SNP pipeline: an automated method for constructing snp matrices from next-generation sequence data. PeerJ Comput Sci 1:e20. doi: 10.7717/peerj-cs.20

[ref12] De Coster W. D’Hert S. Schultz D. T. Cruts M. Van Broeckhoven C. (2018). NanoPack: visualizing and processing long-read sequencing data. Bioinformatics 34, 2666–2669. doi: 10.1093/bioinformatics/bty149, PMID: 29547981 PMC6061794

[ref13] de Jesus Bertani A. M. Vieira T. Reis A. D. Dos Santos C. A. de Almeida E. A. Camargo C. H.. (2023). Whole genome sequence analysis of the first reported isolate of *Salmonella* Agona carrying blaCTX-M-55 gene in Brazil. Sci. Rep. 13:2299. doi: 10.1038/s41598-023-29599-5, PMID: 36759682 PMC9911770

[ref14] Duc V. M. Shin J. Nagamatsu Y. Fuhiwara A. Toyofuku H. Obi T.. (2020). Increased *Salmonella* Schwarzengrund prevalence and antimicrobial susceptibility of *Salmonella enterica* isolated from broiler chickens in Kagoshima prefecture in Japan between 2013 and 2016. J. Vet. Med. Sci. 82, 585–589. doi: 10.1292/jvms.20-0096, PMID: 32213751 PMC7273603

[ref15] Fabrega A. Vila J. (2013). *Salmonella enterica* serovar typhimurium skills to succeed in the host: virulence and regulation. Clin. Microbiol. Rev. 26, 308–341. doi: 10.1128/CMR.00066-12, PMID: 23554419 PMC3623383

[ref16] Ginns C. A. Benham M. L. Adams L. M. Whithear K. G. Bettelheim K. A. Crabb B. S.. (2000). Colonization of the respiratory tract by a virulent strain of avian *Escherichia coli* requires carriage of a conjugative plasmid. Infect. Immun. 68, 1535–1541. doi: 10.1128/IAI.68.3.1535-1541.2000, PMID: 10678971 PMC97312

[ref17] Hallstrom K. McCormick B. A. (2011). *Salmonella* interaction with and passage through the intestinal mucosa: through the Lens of the organism. Front. Microbiol. 2:88. doi: 10.3389/fmicb.2011.00088 21747800 PMC3128981

[ref18] Han J. Lynne A. M. David D. E. Tang H. Xu J. Nayak R.. (2012). DNA sequence analysis of plasmids from multidrug resistant *Salmonella enterica* serotype Heidelberg isolates. PLoS One 7:e51160. doi: 10.1371/journal.pone.0051160, PMID: 23251446 PMC3519518

[ref19] Jacoby G. A. Han P. (1996). Detection of extended-spectrum beta-lactamases in clinical isolates of *Klebsiella pneumoniae* and *Escherichia coli*. J. Clin. Microbiol. 34, 908–911. doi: 10.1128/jcm.34.4.908-911.1996, PMID: 8815106 PMC228915

[ref20] Johnson T. J. Siek K. E. Johnson S. J. Nolan L. K. (2006). DNA sequence of a ColV plasmid and prevalence of selected plasmid-encoded virulence genes among avian *Escherichia coli* strains. J. Bacteriol. 188, 745–758. doi: 10.1128/JB.188.2.745-758.2006, PMID: 16385064 PMC1347294

[ref21] Johnson T. J. Thorsness J. L. Anderson C. P. Lynne A. M. Foley S. L. Han J.. (2010). Horizontal gene transfer of a ColV plasmid has resulted in a dominant avian clonal type of *Salmonella enterica* serovar Kentucky. PLoS One 5:e15524. doi: 10.1371/journal.pone.0015524, PMID: 21203520 PMC3008734

[ref22] Khajanchi B. K. (2022). Foley SL: antimicrobial resistance and increased virulence of *Salmonella*. Microorganisms 10:829. doi: 10.3390/microorganisms10091829 36144431 PMC9504589

[ref23] Khajanchi B. K. Han J. Gokulan K. Zhao S. Gies A. Foley S. L. (2016). Draft genome sequences of four *Salmonella enterica* strains isolated from Turkey-associated sources. Genome Announc. 4:16. doi: 10.1128/genomeA.01122-16, PMID: 27738037 PMC5064110

[ref24] Khajanchi B. K. Hasan N. A. Choi S. Y. Han J. Zhao S. Colwell R. R.. (2017). Comparative genomic analysis and characterization of incompatibility group FIB plasmid encoded virulence factors of *Salmonella enterica* isolated from food sources. BMC Genomics 18:570. doi: 10.1186/s12864-017-3954-5, PMID: 28768482 PMC5541697

[ref25] Khajanchi B. K. Kaldhone P. R. Foley S. L. (2019b). Protocols of conjugative plasmid transfer in *Salmonella*: plate, broth, and filter mating approaches. Methods Mol. Biol. 2016, 129–139. doi: 10.1007/978-1-4939-9570-7_12, PMID: 31197715

[ref26] Khajanchi B. K. Yoskowitz N. C. Han J. Wang X. Foley S. L. (2019a). Draft genome sequences of 27 *Salmonella enterica* Serovar Schwarzengrund isolates from clinical sources. Microbiol Resour Announc 8:18. doi: 10.1128/MRA.01687-18, PMID: 30938709 PMC6430326

[ref27] Liu Y. Y. He D. D. Zhang M. K. Pan Y. S. Wu H. Yuan L.. (2021). The formation of two hybrid plasmids mediated by IS26 and Tn6952 in *Salmonella enterica* serotype Enteritidis. Front. Microbiol. 12:676574. doi: 10.3389/fmicb.2021.676574, PMID: 34122390 PMC8193513

[ref28] Nair D. V. T. Venkitanarayanan K. Kollanoor Johny A. (2018). Antibiotic-resistant *Salmonella* in the food supply and the potential role of antibiotic alternatives for control. Food Secur. 7:167. doi: 10.3390/foods7100167 PMC6210005 30314348

[ref29] Ochman H. Soncini F. C. Solomon F. Groisman E. A. (1996). Identification of a pathogenicity island required for *Salmonella* survival in host cells. Proc. Natl. Acad. Sci. U. S. A. 93, 7800–7804. doi: 10.1073/pnas.93.15.7800, PMID: 8755556 PMC38828

[ref30] Scallan E. Hoekstra R. M. Angulo F. J. Tauxe R. V. Widdowson M. A. Roy S. L.. (2011). Foodborne illness acquired in the United States—major pathogens. Emerg. Infect. Dis. 17, 7–15. doi: 10.3201/eid1701.P11101, PMID: 21192848 PMC3375761

[ref31] Sopovski D. Commichaux S. Zhao S. Grim C. J. Foley S. L. Khajanchi B. K. (2024). Complete genome sequences of 17 *Salmonella enterica* serovar Schwarzengrund isolates carrying an IncFIB-IncFIC(FII) fusion plasmid. Microbiol Resour Announc 13:e0106223. doi: 10.1128/mra.01062-23, PMID: 38231183 PMC10868266

[ref32] Tate H. Hsu C. H. Chen J. C. Han J. Foley S. L. Folster J. P.. (2022). Genomic diversity, antimicrobial resistance, and virulence gene profiles of Salmonella Serovar Kentucky isolated from humans, food, and animal ceca content sources in the United States. Foodborne Pathog. Dis. 19, 509–521. doi: 10.1089/fpd.2022.0005, PMID: 35960531

[ref33] Taylor T. L. Volkening J. D. DeJesus E. Simmons M. Dimitrov K. M. Tillman G. E.. (2019). Rapid, multiplexed, whole genome and plasmid sequencing of foodborne pathogens using long-read nanopore technology. Sci. Rep. 9:16350. doi: 10.1038/s41598-019-52424-x, PMID: 31704961 PMC6841976

[ref34] van der Heijden J. Finlay B. B. (2012). Type III effector-mediated processes in *Salmonella* infection. Future Microbiol. 7, 685–703. doi: 10.2217/fmb.12.49, PMID: 22702524

[ref35] Waters V. L. Crosa J. H. (1991). Colicin V virulence plasmids. Microbiol. Rev. 55, 437–450. doi: 10.1128/mr.55.3.437-450.1991, PMID: 1943995 PMC372828

[ref36] Weinberg E. D. (1978). Iron and infection. Microbiol. Rev. 42, 45–66. doi: 10.1128/mr.42.1.45-66.1978, PMID: 379572 PMC281418

[ref37] Wick R. R. Judd L. M. Gorrie C. L. Holt K. E. (2017). Unicycler: resolving bacterial genome assemblies from short and long sequencing reads. PLoS Comput. Biol. 13:e1005595. doi: 10.1371/journal.pcbi.1005595, PMID: 28594827 PMC5481147

[ref38] Wooley R. E. Gibbs P. S. Dickerson H. W. Brown J. Nolan L. K. (1996). Analysis of plasmids cloned from a virulent avian *Escherichia coli* and transformed into *Escherichia coli* DH5 alpha. Avian Dis. 40, 533–539. doi: 10.2307/1592260, PMID: 8883780

[ref39] Yoon S. Lee Y. J. (2022). Molecular characteristics of ESBL-producing *Escherichia coli* isolated from chickens with colibacillosis. J. Vet. Sci. 23:e37. doi: 10.4142/jvs.21105, PMID: 35332711 PMC9149503

[ref40] Zankari E. Hasman H. Cosentino S. Vestergaard M. Rasmussen S. Lund O.. (2012). Identification of acquired antimicrobial resistance genes. J. Antimicrob. Chemother. 67, 2640–2644. doi: 10.1093/jac/dks261, PMID: 22782487 PMC3468078

