# Supplementary figures and images for: Genetic relatedness and virulence potential of Salmonella Schwarzengrund strains with or without an IncFIB-IncFIC(FII) fusion plasmid isolated from food and clinical sources
Source: Front Microbiol. 2024 May 17;15:1397068. doi: 10.3389/fmicb.2024.1397068 (PMC11143878; doi:10.3389/fmicb.2024.1397068)

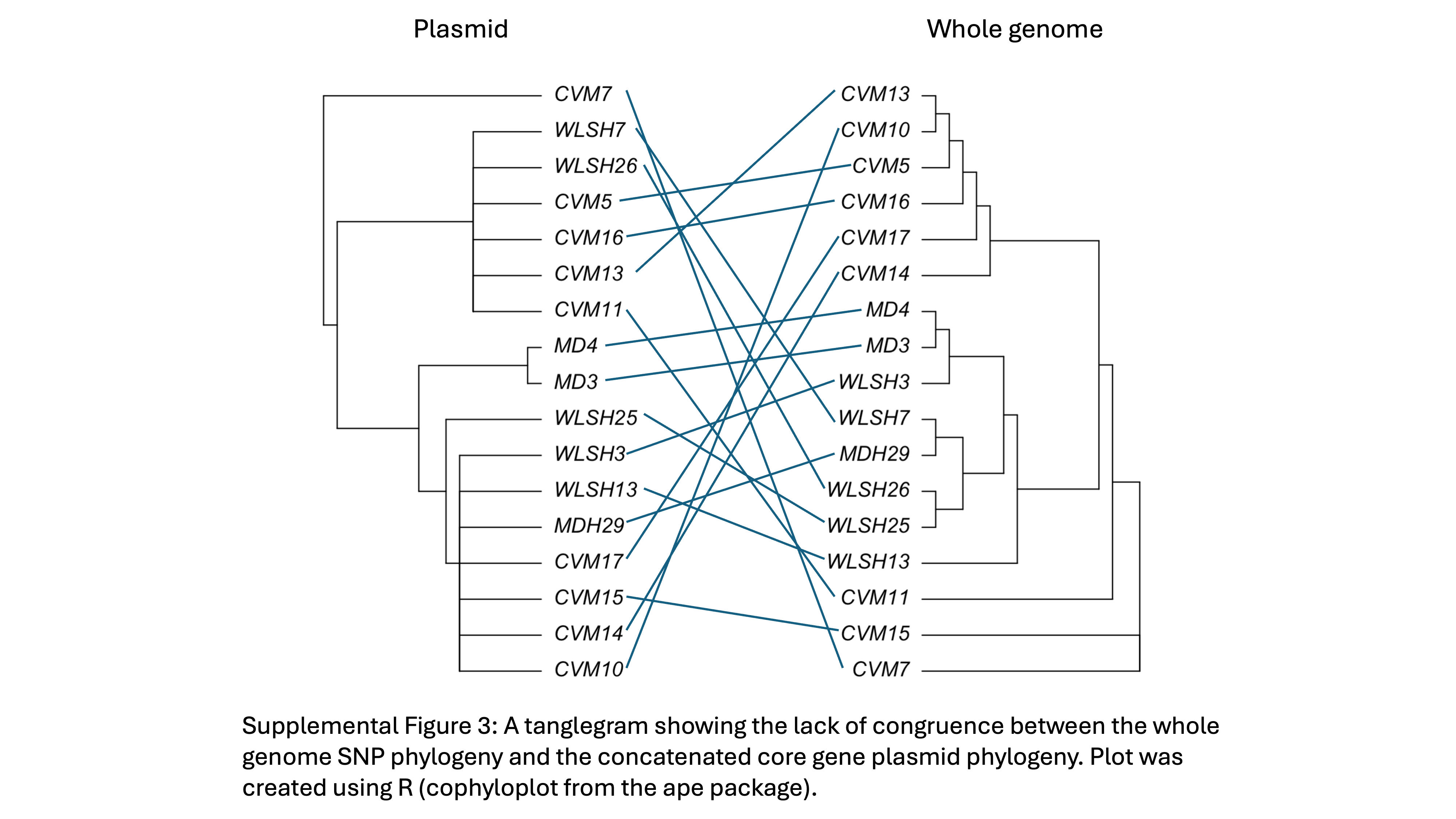

Supplement: Supplementary file 1 [file Image_1.PNG]
