# Supplementary material for: Genetic relatedness and virulence potential of Salmonella Schwarzengrund strains with or without an IncFIB-IncFIC(FII) fusion plasmid isolated from food and clinical sources
Source: Front Microbiol. 2024 May 17;15:1397068. doi: 10.3389/fmicb.2024.1397068 (PMC11143878; doi:10.3389/fmicb.2024.1397068)
Supplement: Supplementary file 2 [file Presentation_1.pptx]

## Slide 1
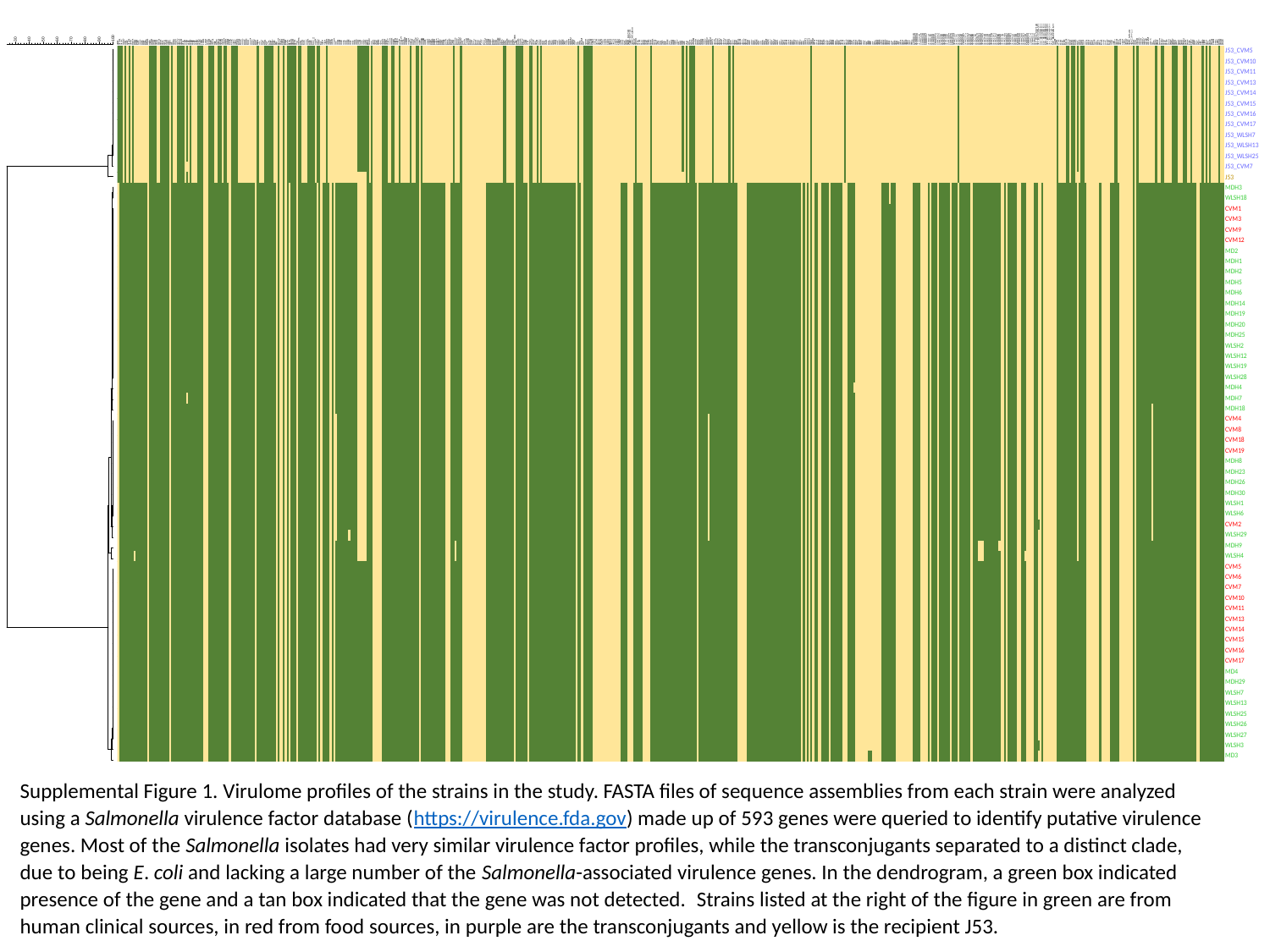

Supplemental Figure 1. Virulome profiles of the strains in the study. FASTA files of sequence assemblies from each strain were analyzed using a Salmonella virulence factor database (https://virulence.fda.gov) made up of 593 genes were queried to identify putative virulence genes. Most of the Salmonella isolates had very similar virulence factor profiles, while the transconjugants separated to a distinct clade, due to being E. coli and lacking a large number of the Salmonella-associated virulence genes. In the dendrogram, a green box indicated presence of the gene and a tan box indicated that the gene was not detected. Strains listed at the right of the figure in green are from human clinical sources, in red from food sources, in purple are the transconjugants and yellow is the recipient J53.
