# Supplementary material for: Genetic relatedness and virulence potential of Salmonella Schwarzengrund strains with or without an IncFIB-IncFIC(FII) fusion plasmid isolated from food and clinical sources
Source: Front Microbiol. 2024 May 17;15:1397068. doi: 10.3389/fmicb.2024.1397068 (PMC11143878; doi:10.3389/fmicb.2024.1397068)
Supplement: Supplementary file 3 [file Presentation_2.pptx]

## Slide 1
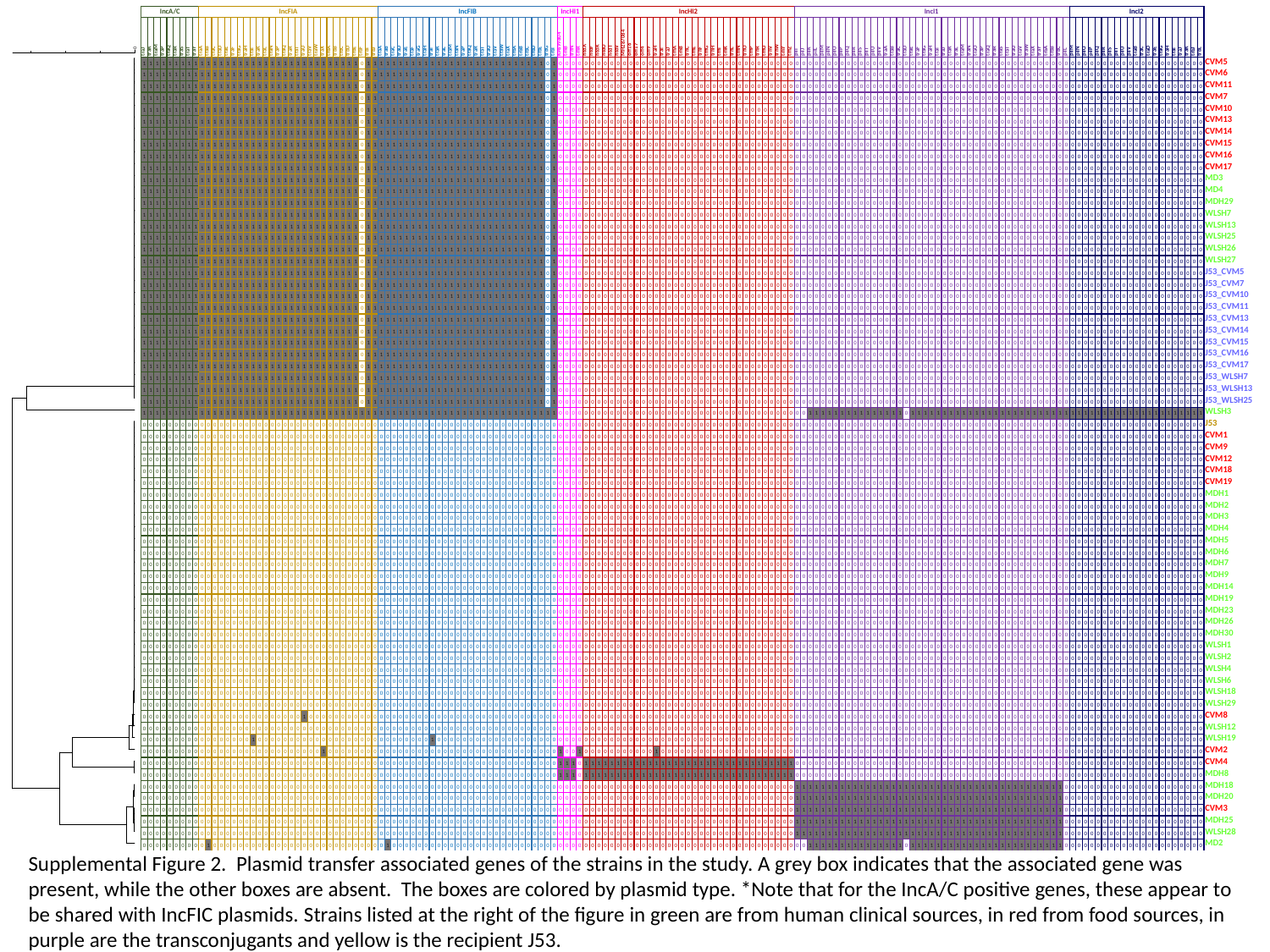

Supplemental Figure 2. Plasmid transfer associated genes of the strains in the study. A grey box indicates that the associated gene was present, while the other boxes are absent. The boxes are colored by plasmid type. *Note that for the IncA/C positive genes, these appear to be shared with IncFIC plasmids. Strains listed at the right of the figure in green are from human clinical sources, in red from food sources, in purple are the transconjugants and yellow is the recipient J53.
